# Role of AI and Histopathological Images in Detecting Prostate Cancer: A Survey

**DOI:** 10.3390/s21082586

**Published:** 2021-04-07

**Authors:** Sarah M. Ayyad, Mohamed Shehata, Ahmed Shalaby, Mohamed Abou El-Ghar, Mohammed Ghazal, Moumen El-Melegy, Nahla B. Abdel-Hamid, Labib M. Labib, H. Arafat Ali, Ayman El-Baz

**Affiliations:** 1 Computers and Systems Department, Faculty of Engineering, Mansoura University, Mansoura 35511, Egypt; sarah.aiyad@gmail.com (S.M.A.); nahla_bishri@mans.edu.eg (N.B.A.-H.); labibm@hotmail.com (L.M.L.); h.arafat_ali@mans.edu.eg (H.A.A.); 2 BioImaging Laboratory, Bioengineering Department, University of Louisville, Louisville, KY 40292, USA; mohamed.shehata@louisville.edu (M.S.); ahmed.shalaby@louisville.edu (A.S.); 3 Department of Radiology, Urology and Nephrology Center, Mansoura University, Mansoura 35516, Egypt; maboelghar@yahoo.com; 4 Department of Electrical and Computer Engineering, College of Engineering, Abu Dhabi University, Abu Dhabi 59911, United Arab Emirates; mohammed.ghazal@adu.ac.ae; 5 Department of Electrical Engineering, Assiut University, Assiut 71511, Egypt; moumen@aun.edu.eg

**Keywords:** prostate cancer, image processing, histopathology images, digital image analysis, computational pathology, artificial intelligence

## Abstract

Prostate cancer is one of the most identified cancers and second most prevalent among cancer-related deaths of men worldwide. Early diagnosis and treatment are substantial to stop or handle the increase and spread of cancer cells in the body. Histopathological image diagnosis is a gold standard for detecting prostate cancer as it has different visual characteristics but interpreting those type of images needs a high level of expertise and takes too much time. One of the ways to accelerate such an analysis is by employing artificial intelligence (AI) through the use of computer-aided diagnosis (CAD) systems. The recent developments in artificial intelligence along with its sub-fields of conventional machine learning and deep learning provide new insights to clinicians and researchers, and an abundance of research is presented specifically for histopathology images tailored for prostate cancer. However, there is a lack of comprehensive surveys that focus on prostate cancer using histopathology images. In this paper, we provide a very comprehensive review of most, if not all, studies that handled the prostate cancer diagnosis using histopathological images. The survey begins with an overview of histopathological image preparation and its challenges. We also briefly review the computing techniques that are commonly applied in image processing, segmentation, feature selection, and classification that can help in detecting prostate malignancies in histopathological images.

## 1. Introduction

Prostate cancer is one of the most common cancers all over the world and considered the second cause of cancer deaths in several countries [1,2]. Nearly one in seven men will be identified to have prostate cancer throughout his life [3,4]. In recent times, statistics show the number of new patients only identified in the United States for 2021 with prostate cancer is nearly 248,530 and the number of deaths is nearly 34,130 [5], so prostate cancer represents a serious healthcare problem in the United States as in many countries. Most tumors do not induce serious clinical symptoms, hence early detection, and localization of prostate cancer at a curable stage is significant for making a medical decision in men with prostate cancer [6].

**Figure 1:**
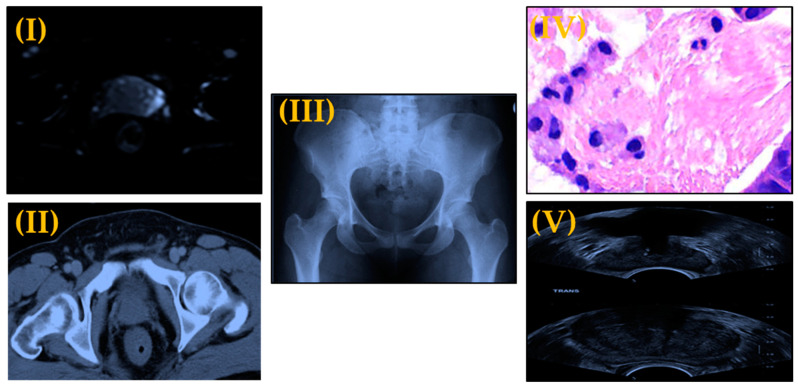
Different Types of Medical Images (**I**) MRI image of prostate, (**II**) CT image of prostate, (**III**) X-Ray image of prostate pelvic area, (**IV**) Histopathological image of prostate tissue, and (**V**) Ultrasound for prostate biopsy.

Because of the lack of progress in the medical field, prostate cancer is increasing as one of the most endemic diseases in the world. The large developments in computing technologies and hardware abilities offer the capability of using computing to tackle issues in many areas. The medical domain is one such area where nowadays a judicious use of technology can assist in improving people’s health and to help in tasks including diagnosis. Medical imaging techniques such as computed tomography (CT), X-rays, magnetic resonance imaging (MRI) and ultrasound imaging (sonography) are great models of computing applications reliant on images, some examples of medical images are displayed in Figure 1. In addition to all of these types of images, histopathology images (HI) are another type of medical image that considered a golden standard to detect cancer and we will focus on it in this survey. HI can be obtained by tissue microscopy from biopsies that help pathologists analyze the characteristics of tissues in a cell basis and study cancer growth [7]. In recent years, many studies have been conducted to capture the entire slide with a scanner and save it as a digital image [8]. The word histopathology derives from the Greek *histos* (web [in this case, of tissue]), *pathos* (suffering or disease), and *logos* (study) [9].

In recent years, computer-aided diagnosis (CAD) has become the main player in radiological detection, diagnosis, and management of diseases [8,10]. Nowadays, computer-aided diagnosis has become a factor of common clinical diagnosis procedures for cancer detection through the use of histopathological images at medical centers and consequently it has become one of the most major topics in histopathological imaging and diagnosis process [11]. There is a substantial requirement for CAD systems to reduce human errors. Human errors happen because of many reasons including lack of expertise or errors caused from image overlapping, blurring, noise, and weak edge detection. Furthermore, observation of the cells specifically composed of visualizing tiny structures, functions, composition, cellular distribution, and cellular morphology across the tissue, which assists pathologists to make a decision of whether the cells are normal and cancerous [11]. This manual process is very time-consuming, difficult, requires a great deal of experience, and leads to variability in diagnosis. Therefore, CAD is a good choice for pathologists for the development in the improvement of histopathological image precision, segmentation of tumor parts, and classification of disease [11]. The literature shows a plethora of CAD systems applied to histopathological images.

In general, artificial intelligence (AI) has shown a significant growth in medical health applications and in histopathology imagery provides a breeding ground for the expansion of CAD systems [12]. AI and CAD systems will continue to grow among researchers and clinicians to constitute a prognostic set of tools to enable them to detect patients that are susceptible to a specific disease and provide accurate, cheap, and fast technologies [12,13]. AI is an umbrella term encompassing both traditional machine learning (ML), and deep learning (DL). The research we consider in our study is largely categorized as ML-based techniques and DL-based techniques. Conventional machine learning techniques applied in HI analysis typically involve several preprocessing steps, including feature selection, image segmentation and classification. ML techniques have been reviewed extensively in the literature, for instance in [2,14,15,16,17,18,19,20,21,22]. In the last decade, researchers have turned their focus towards the development of new deep learning techniques as they outperform conventional machine learning techniques in diverse fields and not only HI image analysis. To date, many of these ML techniques have been supplanted by DL, and an abundance of work has evaluated the use of deep learning techniques on HI of prostate cancer [23,24,25,26,27,28,29,30,31,32,33]. Moreover, studies that employ an ensemble of DL techniques and ML techniques gave better results [34]. Table 1 summarizes reviewed papers on prostate cancer detection and diagnosis. One of the main constraints in conventional ML techniques is their training with a limited number of features, which has been overcome in DL techniques where hundreds to thousands of features can be selected from digital images for classification; however, this process requires significant amount of training time [35]. Some of these problems are solved in ensemble techniques as the feature extraction stage is done using pretrained deep networks and samples classified using conventional ML classifiers [35].

**Figure 2:**
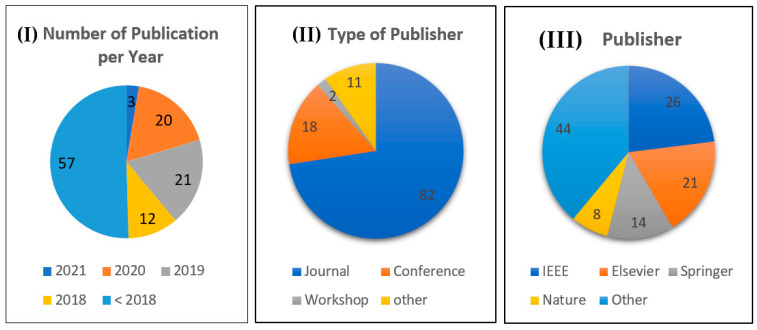
Statistical distribution of studies used in this survey. (**I**) Number of studies per year; (**II**) Type of Publisher, where other denotes a preprint or URL; (**III**) Publisher, where other includes MDPI, Frontiers, AVES, etc.

Many surveys have been published in recent years reviewing histopathological image analysis covering its history, and detailed information of general artificial intelligence techniques [7,8,12,31,36,37,38,39,40,41,42]; the main limitation is the lack of comprehensive surveys of histopathological image analysis that focus on prostate cancer [1,43,44]. Accordingly, in this survey we present more prostate histopathology from an image analysis point of view. The main goal of this survey is providing readers a comprehensive overview of the state-of-the-art in terms of image analysis and artificial intelligence techniques i.e., machine learning, and deep learning being tailored specifically for histopathology images in prostate cancer, and its challenges specific to histopathology images analysis, and the future scope. This survey mentions 113 related works, comprising 63 papers that concentrate on prostate cancer. Figure 2 depicts a statistical distribution of studies used in this survey.

The selection methodology of our survey was conducted using the well-known academic search engines including IEEE Xplore, Google Scholar, Science Direct, Springer, ACM Digital Library, and ResearchGate. We have employed the following criteria: (I) The paper must be highly related to the research area; (II) papers published in highly rank journals and conferences of relevant domain, such as *Scientific Reports, Expert Systems with Applications, IEEE Transactions on Medical Imaging, Neurocomputing, Journal of Pathology Informatics*, etc. and conferences, such as the International Symposium on Biomedical Imaging, IEEE International Symposium on Biomedical Imaging, International Conference on Machine Vision, etc. (III) Top cited papers are preferred. (IV) Papers that were published within the last 5 years, although we also include papers published before that time if the paper is of high quality. Meanwhile, we ignored many papers that have inadequate criteria including low-quality papers, non-English written papers, and white papers.

This survey is organized as follows: Section 2 introduces a background of histopathology images, their preparation, and challenges. Section 3 focuses on the whole histopathology image analysis methodology and highlights the various methods used for this methodology. Finally, we provide some concluding remarks and present some future possibilities in Section 4.

## 2. Histopathology Images Background

Histopathology is a significant branch of biology that covers the investigation of the cell anatomy and tissues of organisms at a microscopic level by a histopathologist [45]. Histopathological images are very influential for the final decision procedure of effective therapeutics; these images are essential to investigate the status of a certain biological structure and to diagnose diseases like cancer [39,45]. Digital histopathology represents a significant evolution in modern medicine [46]. It often uses machine vision techniques as a basis. Nevertheless, because of the special properties of digital histopathology images and their processing tasks, specific processing approaches are usually needed. In this survey, we describe the application of histopathology image analysis employing machine learning and deep learning techniques.

Uropathologists use different screening methods to determine the various tumor histology in the prostate in a good quality. Typical tissue of prostate incorporates glands and stroma. The gland is the basic anatomical structural unit of the prostate. The stroma is the fibromuscular tissue around glands [14]. Each gland unit consists of a lumen and rows of epithelial layers surrounding the lumen. The stroma keeps the gland units together. When cancer is in high-grade, stroma and lumen are both replaced by epithelial cells [24]. Once the slides are stained using a hematoxylin and eosin (H&E) solution, the nuclei become dark blue and the epithelial layer and stroma become several shades of purple to pink [14].

To date, one of the most effective ways to measure aggressiveness of prostate cancer is using the Gleason grading system [24,43,47]. The Gleason grading system is completely founded on architectural arrangements of prostatic carcinoma, and a substantial parameter to a therapeutic final decision. Gleason grading has five grade groups from grade 1 (G1) to grade 5 (G5), with a grade of G1 refers to tissue with a maximum grade of resemblance to normal tissue and outstanding prognosis, and a grade of G5 refers to poorly differentiated tissue and the worst prediction [24,29]. Artificial intelligence has the ability to improve the quality of Gleason grading. Abundant automated Gleason grading systems were proposed and have led to increased consistency [28,29,30,34,48,49,50,51].

**Figure 3:**
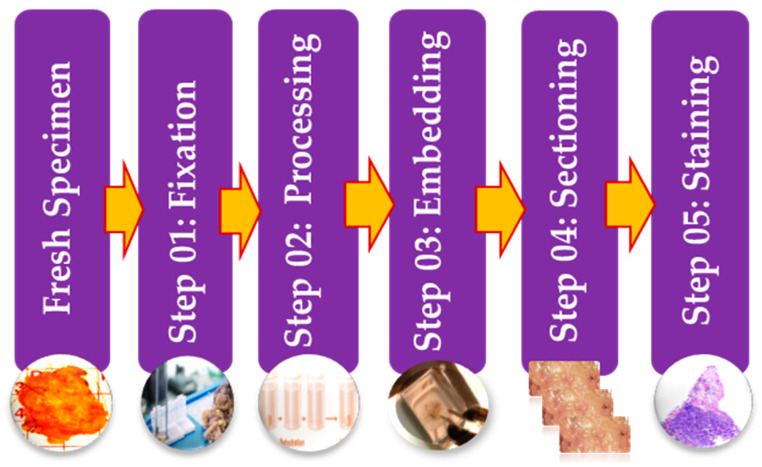
Illustrative figure showing the different preparation steps of histology slides.

Histopathology images can be acquired by using specialized cameras with a microscope wherein an automated computerized approach can be carried out [9]. To study various architecture and constituent of tissues under a microscope, the biopsy specimen is embedded in wax and dyed with one or more stains. Staining procedures are used by pathologists for cellular components separation for structural in addition to component visualization of tissue for diagnosis [38]. Stages of the preparation process of the tissue slides are as presented in Figure 3. It consists of five operations, and each of them can affect the quality of the final image [38,45]. (I) Fixation: Samples of biological tissues are fixed with chemical fixation. There are many ways of fixation, but the commonly applied way in the biomedical field is fixation with formaldehyde or glutaraldehyde solution to protect the cells [51]. This is a critical step in tissue preparation and aims to prevent tissue autolysis and putrefaction; (II) Processing: Tissue processing is a crucial step and involves two main processes: dehydration and clearing. Dehydration is used to extract water from the gross tissue and substitute it with a certain concentration of alcohol which solidifies it [52]. This process helps incise superfine sections of the specimen. Clearing consists of removing the dehydrator with a material that will be the solvent in both the embedding paraffin and the dehydrating agent; (III) Tissue Embedding: Thus is the process wherein tissues are carefully positioned in a medium such as wax [51], so when solidified, it will provide enough external support to allow very thin sectioning. This process is essential as the proper tissue orientation is necessary for precise microscopic evaluation; (IV) Sectioning: this process is required to generate superfine slices of tissue samples sufficient such that the details of the microstructure characterization of the cells can be obviously noticed using microscopy methods. After that, carry the superfine slices of sample onto a clean glass slide [38]; (V) Staining: The final step in preparing tissue for light microscopy is to stain it and mount it on the slide. Staining increases contrast to the tissue and, also highlights some specific features which would otherwise be practically invisible in the microscope [38]. There are many types of stain but the most common type of staining for histology is H & E.

### 2.1. Diagnostic Challenges Using Histopathological Images

Automated prostate cancer diagnosis using histopathology images is deemed to offer great promise for advanced cancer therapy, however, it is not a simple task, as several open scientific challenges have to be overcome before the CAD system of histopathology images can become part of the routine healthcare diagnostic pipeline. These challenges occur because of the numerous technical and computational variabilities and artifacts incurred due to differences in slide preparation and because of the complicated structure of the tumor tissues architecture [41]. Image analysis techniques are substantially reliant on the quality of the digital slide images. In the following paragraphs, we will discuss the different challenges of histopathology image analysis and computational techniques to deal with them.

#### 2.1.1. Extremely Large Image Size

These days, one of the growing challenges is how to handle the extremely large size of histopathology image datasets [53]. Whenever images, for example, cars, humans, or animals are classified using artificial intelligence techniques, small images such as 512 × 512 pixels are usually applied as an input [54,55]. Large-sized images usually have to be rescaled into a smaller size, which is adequate for differentiation, as increasing the size of the input image will result in increased computational complexity, thus making the analysis process more challenging and time-consuming. On the contrary, histopathology images contain as many as hundreds of thousands to millions of pixels, which is generally laborious to analyze as is. Nevertheless, rescaling the whole image to a lower dimension such as 512 × 512 may cause loss of information at the cellular level, which leads to a marked drop of the identification accuracy. Thus, the whole histopathology image is often divided into partial regions of about 1024 × 1024 pixels called patches, where each patch is examined apart, such as detecting region-of-interests [56]. Thus, many studies such as [16,24,25,26,27,48,57,58] presented in this survey, especially those dealing with deep learning applied patching technique to overcome the extremely large histopathological images.

#### 2.1.2. Insufficient Labeled Images

Perhaps the biggest challenge in analyzing histopathological images is that only a limited number of training set data is available. As healthcare image datasets often have a considerably lower size than a natural view of images, this causes direct application of many conventional artificial intelligence techniques not suitable for medical image datasets [53]. One of the important keys of success of DL in common image recognition tasks is the abundance of training data. Label information at a pixel level or a patch level is essential in histopathology image tasks such as diagnosis. Label information could be collected easily in common image processing from the internet and it is also possible to use crowdsourced labeling since the human brain is able to identify objects and perform labelling work while ignoring artifacts [59]. Nevertheless, only highly qualified pathologists can manually label histopathological images properly, and this process at the regional level in a large histopathology image needs a long time and is tedious. Therefore, the paramount limitation in designing high-quality histopathology image analysis techniques lies in the paucity of freely public annotated datasets [24,60]. Many researchers have attempted to alleviate such a problem of insufficient amount labeled images. Most of these solutions fall under one of the following categories: (I) increasing the number of labeled data, such in [25,30], (II) predicting the labels of test images or self-taught learning, such as applying transfer learning [24,61], or (III) utilizing of weak label or unlabeled data [62].

#### 2.1.3. Artifacts and Color Variation

**Figure 4:**
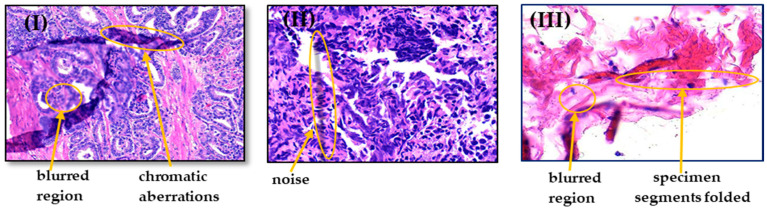
Examples of possible artifacts in histopathological images, where (**I**) contains chromatic aberrations and blurred regions; (**II**) contains noise, and (**III**) contains specimen segments folded and blurred regions.

Another major challenge is the presence of artifacts and color variation [8,11,36,59,63,64]. Histopathology images are captured through several stages as previously mentioned. At each stage, unwanted anomalies that are unassociated with the underlying biological factors, could be represented by differences in specimen preparation, staining, and even scanning with equipment from different vendors. For instance, when specimen sections are placed onto the slides, they may be folded and rumpled; dust may besmear the slides during scanning process; loss of microscope focus leads to blurred regions, noise, and shadows; and occasionally tissue regions are marked by color markers or chromatic aberrations [8,41]. Learning without considering these artifacts, as shown in Figure 4**,** may deteriorate the performance of decision support algorithms. When digital images are produced, the slides should be uniformly illuminated by the light source. Tissue autofluorescence differences in microscopic setup, staining protocol, and organ size could generate irregular lighting across the tissue samples. Additionally, the scanner’s sensitivity varies for different wavelengths of the light spectrum [41]. Large variations in light are considered an important factor for the precise prostate cancer diagnosis. These variations need to be handled earlier before employing image processing techniques [63,64].

To tackle these problems, many different techniques have been designed, including conversion to grayscale [65,66], color normalization [67,68], and color augmentation [69]. One of the simplest methods is the conversion of colored histopathology images to grayscale, however, it disregards the significant information concerning the color representation used by pathologists since the beginning. On the contrary, the color normalization method attempts to adapt the color values of images on a pixel-by-pixel basis so that the color distribution of the source image matches a reference image. Color separation and stain normalization were applied on the histopathology images for the first time in [70]. Afterwards several distinct color and stain normalization techniques have been used as a preprocessing step in several techniques for histopathological image analysis.

#### 2.1.4. Multi-Level Magnification Led to Multi-Level Information

Magnification is the phenomenon of enlarging the proportion of biological structures that are apparent under the microscope based on different objective lenses [39]. Traditional microscopes have a standard set of objectives with 2X, 8X, 40X, 200X, and 400X power [39]. Tissues generally consist of cells and fibers, where each tissue shows specific cellular features. Information concerning cell shapes is taken accurately under a high power objective and images are more deterministic and informative to predict disease outcome, but structural information such as a glandular structure that are made of many cells are better taken under a lower magnification, so that images cover a wider field of view. Because malignant tissues exhibit both cellular and structural abnormalities, each of the images captured at different magnifications could provide significant information. Even in AI, researchers employing image datasets with different levels of magnifications, such as in [71,72]. As already pointed out, it is challenging to process the images at its original resolution directly, images are usually rescaled to adapt different magnifications and configured to be input for processing. Regarding diagnosis, the most informative magnification remains a subject of controversy, whereas efficiency enhancement is sometimes attained by entering both low and high magnification images simultaneously as input, probably depending on the applied AI technique or type of disease. Moreover, the status of histopathological images does not need to be determined by the cells, images with different levels of magnification are adopted to learn distinctive features [71].

**Figure 5:**
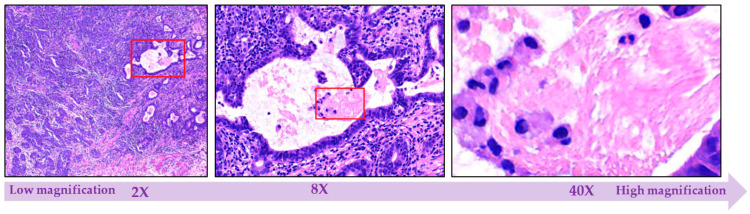
Illustrative figure showing the different levels of magnification (starting from 2× up to more than 40×) that might be applied on histopathological images.

As depicted in Figure 5, histopathological images with multiple levels of magnification can depict various types of information. When the histopathological images are with low magnification, cells will be difficult to detect, while the high magnification image shows more fine-grained details.

## 3. Histopathology Image Analysis Methodology

Digitized histopathology is a current direction that makes huge numbers of images available for automated analysis. It enables visualization and interpretation of pathology cells and tissue samples in a great resolution images and with the assistance of software tools [36,37]. This opens a new era to design image analysis techniques that assist clinicians and promote their image descriptions (e.g., grading, staging) with the purpose of image features quantification. In that respect, the computer-aided diagnosis of histological image analysis is a newly challenging domain for biomedical image analysis. CAD can be defined as detecting cancer within the examined tissue using computer software [60,73,74], which is the main mission of the pathologist [8]. The combination of conventional diagnosis techniques with computational AI techniques provides a good possibility to decrease the workload of pathologists while preserving performance. There is a need for a precise CAD system that minimizes reading interpretation times, lowers necessary experience in anatomic pathology, and provides a consistent risk evaluation of cancer existence in prostate histopathology images without additional burden to pathologists. Such a CAD system would automatically find out suspected lesions in prostate histopathology images to assist screen for prostate cancer in large patient populations. A typical CAD system for detecting prostate cancer receives raw histopathological images, preprocesses them, and produces a particular diagnostic result [10].

Over the last two decades, numerous research papers on CAD systems were published. Automated systems for digital histopathological imaging can maintain reproducibility and consistency using suitable image processing techniques [41]. In fact, there are many research perspectives for CAD systems applied in the histopathological domain, including: (I) cancer detection in the given tissue, (II) automatic grading to correctly quantify the level of the malignancy, which can offer more insights into disease characterization, (III) cell/nuclei/gland segmentation that discovers and separates these regions from images, and (IV) multi-class classification for the different subtypes of a specific type of cancer.

**Figure 6:**
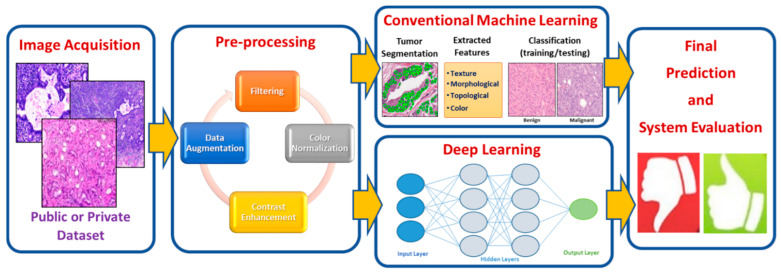
An illustrative block diagram of a typical prostate CAD System starting from the image acquisition until obtaining the final diagnosis.

CAD systems can be broadly subdivided into two groups. The first uses handcrafted features and relies on conventional machine learning techniques, while the second uses deep learning techniques. For this reason, we will discuss these two groups separately in Section 3.2 and Section 3.3, below. Figure 6 displays the process model for handcrafted features based on machine learning techniques versus deep learning techniques of histopathological image analysis. The process model of the two groups of analysis passes through a number of stages that highlight specific structures in the image analysis methodology. There are two common components that are shared by the process model, which are image acquisition and image preprocessing.

### 3.1. Image Acquisition

In the first phase, histopathology images can be acquired from a public dataset or a private dataset. The choice of a dataset is a dominant factor to establish for any experimental setup. One of the main challenges when dealing with prostate histopathology images is the lack of representative public image datasets annotated by multiple pathologists with high quality. Most research dealing with prostate histopathology images work with private datasets. As shown in Table 2, we provide list of the publicly available datasets [75,76,77,78,79]. It is noted that PANDA challenge [78] provides the largest public histopathology image dataset in prostate cancer.

### 3.2. Image Preprocessing

**Figure 7:**
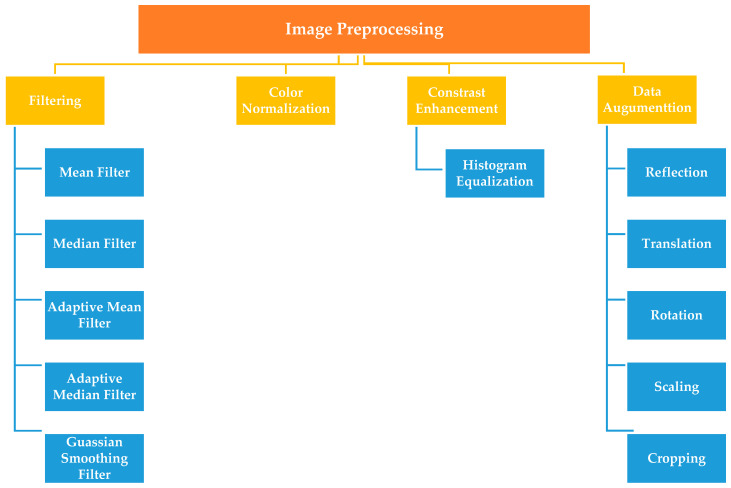
Taxonomy of different image preprocessing methods.

Preprocessing is a basic stage of most automated CAD systems [35]. In the preprocessing stage, raw data are processed to normalize the image or to transform the image to a domain where cancer can be easily diagnosed [10]. Preprocessing can enhance histopathology images and ameliorate the interpretability for human viewers since the acquired images contain different types of noises or artifacts and may not have adequate contrast or illumination due to the scanning [36,46]. It is necessary that the acquired images be of good quality to generate the intended result [40]. Appropriate image pre-processing methods could compensate for these differences between images. Various existing preprocessing methods are commonly used to boost the results of the analysis process can be grouped as illustrated in the following subsections and summarized in Figure 7.

#### 3.2.1. Filtering

There are various methods for enhancing images. The basic and simple methods can be classified as filtering. Filtering is used to eradicate unwanted variation (noise) from images. There are different noise eliminating filters used for removing undesirable information from images, i.e., mean filters, median filters, adaptive mean filters, adaptive median filters, and Gaussian smoothing filters. The mean filter is the simplest linear filter [80]. It eliminates the noise, blur images, and reduces sharp edges [81]. Similarly, the median filter has also been employed to eliminate noise from histopathology images [40]. The median filter is a nonlinear digital filtering method. It is commonly used in digital image processing because under certain conditions, it maintains edges whilst removing noise [82]. Adaptive filtering is used to remove noise from images without degradation. It involves a tradeoff between smoothing efficiency, preservation of discontinuities, and the generation of artifacts. Gaussian filtering is a smoothing filter method. It has been applied for smoothing the images, to overcome the variations in staining, as well to reduce noise [40]. The Gaussian filter is a very good filter for removing noise expressed in a normal distribution [80].

#### 3.2.2. Color Normalization Techniques

In histopathology CAD systems, color normalization plays a significant role because the perception of information in images could negatively affected by color and concentration differences [83,84]. Two issues have made the color normalization process a challenging task [83]: (I) the presence of diagnostically significant but visually subtle details in color images. (II) the heterogeneous nature of tissue composition. Among the image preprocessing techniques, color normalization was the most common. In the last two decades, many color normalization techniques to histopathology image analysis have been proposed. In [85], authors developed a reliable color-based segmentation approach for histological structures that applied image gradients estimated in the LUV color space instead of RGB color space to handle matters relating to stain variability. Another approach presented in [84], founded on using of nine common color filters selected for histology H & E stained slides. The authors conducted two experiments, and results showed that pathologists became more sensitive to the color of the image than before. While in [86], a new color correction technique is proposed and developed in the linear RGB color space. This technique can easily be integrated to the slide scanning process. The technique is also handy in the sense that the data needed for color correction are extracted from the color calibration slide wherein nine reference color patches embedded on the glass slide, and the spectral properties of these patches are known beforehand.

#### 3.2.3. Histogram Equalization

The histogram of an image is a mathematical graph representing frequencies of occurrence of distinct color intensities in that image. It summarizes the image with respect to quality, contrast, and brightness [40]. Histogram equalization of the image is a popular and simple ways for enhancing image contrast to normalize the distribution of probability of occurrence of intensities in the image and used for removing color variations due to illumination conditions and staining process [40]. There are many previous works published in histogram equalization. In [87], the authors tried to overcome the problem of changing the brightness of an image when applying traditional histogram equalization. They introduced a novel extension of bi-histogram equalization technique. It effectively separates the objects from the background. Another novel method for histopathology images was introduced in [88], is a fully automated stain normalization technique to minimize batch effects and thus help improving analysis of digitalized pathology images. Among the different histogram techniques, one paper applied multi-objective histogram equalization by using particle swarm optimization (PSO) [89]. The proposed technique works by segmenting the histogram of the image into two sub-images. Then, a number of optimized constraints are employed. PSO used to explore the optimal constraints. This technique preserves the brightness of the image while enhancing the contrast.

#### 3.2.4. Data Augmentation

In the artificial intelligence domain, the model efficiency always enhances with the amount of the training data that has been used. Data augmentation (DA) is a strategy used to artificially enlarge the size of the training data without introducing labeling costs [90,91,92,93,94]. DA has already been used in many domains, including image processing and audio classification. The most common means of data augmentation in image analysis include reflection, translation, rotation, scaling, and cropping [90]. Applying conventional data augmentation methods is one popular way to increase both the number and diversity of images in small datasets. Nevertheless, it is not always used in all problems. A significant amount of DA techniques on specific problem-dependent are proposed can also be applied to expand small datasets. One of the powerful and common methods used in data augmentation is generative adversarial networks (GANs) [91]. GANs are based on competition between two neural networks. GANs consist of a discriminator and a generator, two neural networks trained as adversaries, therefore its name is adversarial. Over the past years, there have been many attempts in exploring the use of GANs in generating synthetic data for data augmentation given limited or imbalanced datasets. One variant of GANs is proposed in [92]. It is used to enhance generalizability in CT segmentation tasks. Another variant of GANs used in histopathology images proposed in [93]. But applying these techniques always require a relatively high effort. Moreover, there exist lots of excellent studies for data augmentation. In [94], the authors proposed a novel technique capable of augmenting histopathology images and distributing the variance between patients through image blending using the Gaussian-Laplacian pyramid. This technique produces new training images composed of half images of different patients. This method tries to prevent that a model learns color representations of patients, which related but to the staining process. Some studies aim to enhance the overfitting problem caused by the lack of samples by employing different data augmentation techniques. For example, in [26] authors used five DA techniques (rotation, flipping, shifting, rescaling, and random elastic transformation). Experimental results showed the effectiveness of applying different DA methods in the nuclei segmentation task.

### 3.3. Traditional Machine Learning Techniques

Machine learning (ML) is an automated learning process of machines to categorize and recognize different data such as text, images, and videos. ML employs algorithmic techniques to analyze, learn, and make decisions from the input data [95]. ML has been widely employed in many applications, including image processing, specifically in our study in histopathological image analysis. Traditional machine learning techniques typically involve several steps to deal with histopathology images including segmentation, feature extraction, and classification, as represented in Figure 6. Each step is described in the following subsections.

#### 3.3.1. Image Segmentation

Segmentation process is one of the main research efforts in histopathology image analysis. It is the process of separating objects in an image that are of interest to the developed application by using various methods [40]. It can make anatomical structures like glands, nuclei and so on more obvious for a subsequent automatic or manual image classification [7]. The various morphological features of these structures like size, shape, extent, and color intensity, are also important factors for existence of prostate cancer. To analyse all these indicators, images need to be segmented first [38]. Prostate segmentation is a challenging process. It is difficult to determine the boundary between the prostate and the surrounding tissues. Even for experienced pathologists, the interobserver variability of manual prostate segmentation is large [10]. A precise prostate cancer segmentation may help effectively in guiding radiation therapy and biopsy therapy as well as its application in diagnosis [10].

**Figure 8:**
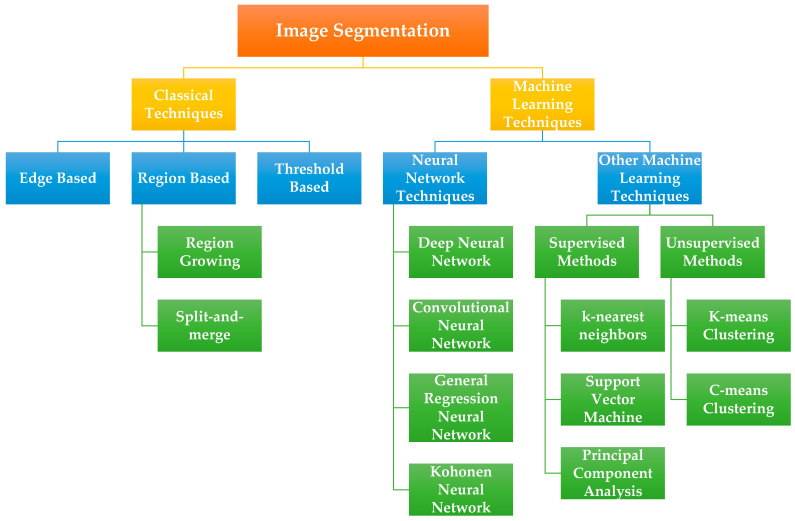
Image segmentation taxonomy compromising different techniques that are used to segment histopathological images.

Many researchers have applied various segmentation techniques in their research, which can be broadly classified into classical techniques and machine learning techniques, as represented in Figure 8. However, there is no general segmentation technique proven to be effective for all kind of images. In [23], the segmentation task in prostate cancer is carried out using the color space transformation and thresholding techniques. This process aids to form the gland region, which is subjected to feature extraction by applying multiple-kernel scale-invariant feature transform method. In [15], authors presented a new automatic nuclei and gland segmentation technique for prostate histopathology which incorporates an integration of high-level, low-level, and domain-specific information. The segmentation technique is utilized for three different applications: (I) classifying intermediate grades of prostate cancer, (II) identifying cancer from normal regions, and (III) discriminating Bloom-Richardson high-grade cancer from low-grade cancer. In [16], authors proposed an automated technique for gland segmentation in prostate cancer using histopathology images using machine learning and image processing methods. This technique outperforms structure and texture-based techniques. However, this technique fails in the images with the cribriform pattern, resulting in inaccurate segmentation. Another study [96] tried to overcome the necessary condition of the conventional thresholding segmentation method to give accurate results, where the nuclei must have a wide range of intensities to be easy differentiated from the background. Their adaptive thresholding technique passes through four different stages: (I) detecting the nuclei, (II) optimizing the primary contours through a rough texture segmentation, (III) optimizing the convergence, and finally (IV) splitting the overlapping segmentation masks.

Other methods such as [17] used two-stage segmentation. Firstly, the mean-shift (MS) algorithm is used to perform the coarse segmentation to split the tissue constituents in four parts. After that, wavelet filters are used to perform fine segmentation of glandular tissue. Although, there exists other studies that segment each individual cell. for example, an early study [97], where authors focused on dynamic segmentation of live cells for the purpose of quantification of different modalities. Their technique can identify the cell boundary no matter how many times it is used in the system.

There exist few studies that focus on utilizing cell nucleus and blue mucin. In [98], authors depend in their segmentation on the structure of glands to separate them from the background by analyzing the color space of histopathology image. Another segmentation technique, proposed in [99], combined the similarity of morphological characteristics related to the appearance of lumen components. It operated in three stages: (I) classification of pixels, (II) extraction of inner gland boundary, and finally (III) complete gland construction. The performance of the abovementioned techniques is constrained by the size and the characteristics of labelled datasets and the variation needed in the images to model the distribution of relevant tissue features.

#### 3.3.2. Feature Selection

Feature selection refers to eliciting the best feature subset that can accurately label images from a dataset as belonging to one or more classes [100,101]. This has now been a significant domain to researchers with new advancements in histopathological image analysis. Just a few applications produce their data already in a form that classifiers can construe and do not need a feature selection process. However, histopathology images require representing characteristics of the tumor cells or tissues in a quantitative way [7,41]. The extracted features should be identifiable and distinct to an extent to be able to automatically classify normal and malignant tissues and to grade them correspondingly [41]. In HI, selecting which distinctive features will be feeding the classifier is more essential than picking the classifier itself, and when feature selection is applied, classification accuracy will be improved as many features are selected from all features [10]. Selecting distinctive features from targets of interest is a challenging task in an effective CAD system. Common features for HI comprise size, shape, histogram, texture, intensity, and multiple features. Feature descriptors to be selected in HI can be categorized into four groups: texture-based features, topological-based features, morphological-based features, color-based features, and other features [38,39,45,46]. Table 3 provides a brief view for the feature extraction publications suggested in HI of prostate cancer. The following paragraphs detail the different features selection procedures that have been employed for classifying histological images.

Texture-based features are related to the spatial distribution of repetitive intensities inside the tissue [9]. Examination texture features of each tissue components gives a valuable discriminative information in the diagnosis and grading systems of prostate cancer. In [56], authors applied a quantitative texture feature selection, for example, gland density, gland size, and gland circularity, and evaluated the accuracy of these features in discriminating normal from cancer glands using the ROC curve. The model achieved an average of 0.94 of AUC. In [102], a new method was proposed to overcome redundancy among features and that considered one of the most important reasons for weakness of SVM-RFE. The main purpose of their proposed feature selection method is to merge the SVMRFE with filter measure to extract the least features and enhance the classification accuracy of the model. Another work [103] focused on a type of texture-based features, called local binary pattern (LBP), and introduced a new modified version called multispectral multiscale LBP (MMLBP). This algorithm varies from the standard LBP in which it takes into consideration the joint information within spectral and spatial directions of the image. MMLBP attained a classification accuracy of around 99%.

Topological-based features enable characterization of cellular structure in histopathology images. These features apply the theories of algebraic topology and this is especially beneficial to the segmentation task [13,39]. In [13], 50 topological-based features were selected for designing a new data fusion algorithm in prostate histopathology images, incorporating 25 nearest-neighbor and 25 graph-based features. A pioneering effort on the use of topological features for automated scoring of prostate cancer using histopathological images was done in [50], where the authors introduced a new class of topological features that make use of network cycle structure. Another work [49] selected a set of visually significative features for the purpose of differentiation between different grades in prostate cancer using topological-based features. It based on computing the shortest path from the nuclei to their closest luminal spaces.

Morphological-based features give information about shape, color, structure, and size of the cells in HI [39]. Morphological features are useful to provide details for form and structure of abnormal cells of prostate cancer [9]. Many studies showed the viability of this type of features to help characterization of the histopathological prostate images. In [15], they presented a new automatic gland and nuclei segmentation system for prostate histopathology images and utilize an accurate extraction of various morphological features. In [104], the authors presented a content-based image retrieval system that takes advantage of a novel set of morphological attributes called explicit shape descriptors that properly depict the similarity between the morphology of objects of interest. A recent study [58], proposed a new machine learning classification method to classify Gleason grade groups of histopathology images for prostate cancer using new proposed morphological features.

Color-based features provide information of the grey level or color of pixels provided in the region of interest. Feature selection based on this type of features utilizes different color spaces. In [98], authors introduced a novel technique for grading prostate malignancy using digitized histopathological specimens of the prostate tissue. The color space that represents the tissue image is the Lab color space. The Lab color space is preferable than RGB since it is designed to approximate the color perception in human visual system. Also, in [14] classification is based on the lab color space. In [105], authors presented a wavelet-based color feature selection technique utilizing CIELAB color space. They compared CIELAB in their experiments with many color spaces e.g., RGB, KLT and HSV. CIELAB attained the highest accuracy.

However, most of the research that focus on feature selection apply a combination of different types of feature selection to improve the performance. The work presented in [106] introduced a new content-based microscopic image. The authors applied a hybrid color and texture feature selection method. They used RGB and HSV color spaces for color-based feature selection and for each image, an overall of 80 texture features were selected. The performance of the retrieval system was evaluated for various histopathology image types and the best retrieval performance was obtained for prostate images. In [107], the authors proposed an integrated feature set that combines color and morphological features to design new CAD system to automatic grade prostatic carcinoma biopsy images. Another CAD system was introduced in [50] to automatic grade of prostate cancer. The research used a total of 102 topological-based, morphological-based, and texture-based selected features from each tissue patch so that quantifying the arrangement of glandular and nuclei structures within histopathological images of prostate cancer tissues. Another recent research in [27], provided an automatic system able to accurately detect specific areas susceptible to be cancerous through presenting a novel method, a combination of topological-based, morphological-based, and texture-based feature selection for addressing the hand-crafted feature selection stage.

#### 3.3.3. Classification

Classification is one of the important data analysis domains, which focuses on assigning a sample to one of a set of classes, based on its features [108,109]. For histopathological images, choosing the appropriate classifier is very significant to cope with huge, high visual complexity datasets. After segmentation and feature selection, the selected optimal classifier is applied to classify images for detecting malignancy in HI. In this step, a cell or tissue is assigned to one of the classes and then it can also be classified for malignancy level e.g., grading of tumor or type of the tumor [38]. Machine learning classifiers operate in two modes: learning mode and classification mode. In the learning mode, the selected features from annotated histopathological images are used to train the classifier. Afterwards, the classifier is used in classification mode on cases without knowledge of true annotation [10,41]. The different selected features from HI are used to classify the new images as normal or malignant. Constructing automated classifier systems of histopathological images is a challenge task in machine learning as histopathological images do not hold the same morphologic structure of macroscopic images such as human faces, trucks, text, or animals [94]. Numerous classification methods have been developed for histopathological images employing machine learning algorithms like k-nearest neighbors (KNN), support vector machine (SVM), logistic regression method, random forests (RF), decision trees, fuzzy systems, etc. The details regarding the developed classifiers dealt with classifying histopathological prostate images have been summarized in Table 4.

KNN is one of the simplest, versatile, and efficient methods used for image classification [99]. For instance, the authors in [66] applied KNN to classify HI into four grades of cancer ranked from 2 to 5. They used different K, e.g., 1, 3, 5, 7 and compared the results. With K = 1, achieved the highest performance of classification. Another work [18] applied a KNN classifier with K = 3 to develop an analytical framework to differentiate between stroma and glands in histopathological images of radical prostatectomies and to differentiate different Gleason grades. The proposed framework can be used firstly before quantifying and stratifying anatomic tissue structures.

In theory, a support vector machine (SVM) algorithm could obtain a high performance because it can maximize the margin between normal and cancerous training samples [10]. There exist many works that make use of SVM classifiers in prostate cancer histopathological images [13,14,15,48,58,103,106,107]. In [14], a novel methodology was proposed for labelling individual glands as normal or cancerous. They applied SVM classifier. SVM is trained by a linear kernel function to filter out the non-nuclei objects. In [13], the authors addressed the classification stage using a hand-crafted method that make use of two widely known classifiers. Specifically, they optimized SVM classifier and used a quadratic kernel to handle the multi-class classification from a nonlinear method. They achieved promising results. In [58], the authors developed an automated grading system for histopathological images of prostate cancer using SVM. After several experiments to compare between SVM and multilayer perceptron classification method (MLP), they reached to that SVM attained better results than MLP. Another study introduced a new system for quantitative and automated grading of prostate biopsy samples [48]. This work used a SVM classifier to differentiate between four categories of tissue patterns and they used cross-validation to get the best parameters for the classifier.

Inspired by the bag-of-words (BoW) model extensively used in natural language processing, the authors in [22] developed a new CAD system for prostate cancer using speeded-up robust features (SURF). In [21], a new method named multi-level learning architecture (MLA) is proposed. It depends on the divide-and-conquer algorithm by assigning each binary task into two different subtasks e.g., (strong and weak).

Multi-classifier systems or ensemble-based combine accuracies of different similar classifiers for improving the predictions for a problem [7,36]. Early research [20] employed a modified version of the popular ensemble classifier AdaBoost. To the best of our knowledge, their research is the first attempt at automatically analyzing prostatic adenocarcinoma across multiple scales. Some researchers tried to propose a classification technique to work in multiclass problems. In [19], another ensemble method (SVM plus random forests) was used to adapt to various imaging modalities, image features, and histological decisions. They employed statistical analysis using the Friedman test to rank the results of classifiers on datasets. To the best of our knowledge [110] is the only example that applied a fuzzy system to HI of prostate cancer, where the authors designed membership functions of the fuzzy system by using a genetic algorithm. In [2], the authors presented an adaptive boosting algorithm to support automated Gleason grading of prostate adenocarcinoma (PRCA). They prepared a pool of classifiers (SVM with linear and radial basis function kernels, adaptive boosting algorithm, decision tree, RF, linear discriminant analysis (DA) and quadratic DA). Results of all classifiers were combined using an adaptive boosting classifier.

### 3.4. Deep Learning-Based Techniques

Recently, adoption of deep learning (DL) techniques in biomedical imaging has had a positive impact on a broad range of tasks including automatic analysis of histopathology images [34,36]. DL creates new clinical tools that outperform the aforementioned classical machine learning techniques with handcrafted features in terms of accuracy, objectivity, consistency, and reproducibility. It also provides new insights to clinicians and researchers [59]. DL techniques are currently the most frequently studied in prostate cancer histopathology imaging and studies [28,34] have proven that DL models can accurately detect cancer in histopathological images. DL techniques takes original digital images as input, with a minimum preprocessing, and have the benefit of learning features instead of the conventional selection of handcrafted features, which may be not sufficient or not accurate [34]. Deep learning techniques learn salient features from data, so a large number of input images is of great value to the training process. Deep learning cannot be regarded as a singular technique; it can nearly be considered as adaptation of multi-layer artificial neural networks to a large variety of challenges, from natural language processing, fraud detection to computer vision [31]. Neural networks consist mainly of an input layer, a number of hidden layers, and an output layer, where each layer is composed of neurons. The input layer firstly takes input data, then the hidden layers execute some mathematical computations on those input data [111]. The output values of the network are predicated on the adjustment of internal weights [36]. These weights are computed by the network through iterative forward or backward propagation of the training data and error backpropagation respectively [36]. This process takes less effort to code than the conventional machine learning.

The main obstacle of any deep learning technique is its need for a substantial training set. Fortunately, histopathology images contain a great deal of information at small scales. Accordingly, a single slide can produce considerable amount of training patches [34]. Patches generate the effect of extracting portions of an image with the same structure but relate to images belonging to different classes [7]. Patches are commonly square portions having dimensionality that ranges from 32 × 32 pixels to 10,000 × 10,000 pixels [59]. Another obstacle of deep learning is the inadequacy of interpreting features and this may slow the development of CAD systems [34]. In the last decade, neural network architectures like convolution neural network (CNN), fully convolutional network (FCN), deep neural networks (DNN), and generative adversarial networks (GAN) are attracting the attention from the research community because of its recently impressed results on large datasets. A considerable amount of effort is done on prostate cancer histopathological images using the different neural networks.

A particular neural network subtype, convolutional neural network; has made sound advancements in image processing [31,112]. Convolutional networks have the ability to identify visual patterns with less processing and is persistent in existence of variations and distortions in pattern [36]. The basic CNN structure is comprised of convolutional, pooling, activation, classification, and fully connected layers [36,90]. The Histopathology imagery domain is rapidly adjusting this architecture to enhance a wide range of challenges. In [31], authors investigated the general applicability of CNN for increasing the performance of prostate and breast cancer detection in histopathology images. They used fully connected CNN to get cancer maps for each pixel and make segmentation in the whole slide images. Results proved that DL has great potential for increasing the performance of detecting malignancies in H & E images as AUC ranges from 0.88 to 0.99. As far as we know, researchers in [54] were the first to use images of the entire prostate gland as an input to the network, instead of using image patches or regions with gland information. They designed a new CNN architecture that comprises feature selection stage, characterized by the compound of four convolutional blocks, and the classification phase compound of two fully connected layers.

Various papers have applied CNN to automatic Gleason grading to perform better than systems that use conventional machine learning methods. The first attempt to apply convolutional networks to Gleason score grading prediction is [30], where the authors applied a pre-trained CNN. The classification stage in CNN was excluded and replaced with RF and SVM algorithms to classify the feature vectors selected from the network. In [28], the authors trained different variants of CNN as Gleason score annotator and utilized the prediction of the model to assign patients into low, medium, and high levels of risk, attaining pathology stratification results at expert level. Their experiments shown improved efficacy regarding the applicability of CNN reaching more reproducible and consistent prostate cancer grading, specifically for cases with heterogeneous Gleason patterns. Recently, a fully automated grading system using the U-Net was proposed in [29], where the authors adopted the conventional U-Net architecture, however after several experiments, they made the network deeper to be composed of six levels as they added additional skip connections within each layer block. Their model attained a high agreement with pathologists.

Aside from CNN, many authors have tried to utilize different techniques in histopathology imagery in prostate cancer, for example, the authors in [23] proposed a new deep learning technique that combines the multi-model neural network, ride NN and optimization algorithm, Salp–Rider algorithm (SRA), generating the new technique SSA-RideNN. The experiments showed that SSA-RideNN attained a maximal accuracy, specificity, and sensitivity.

Since the comparison of different techniques is difficult, some studies like [34] tried to compare different classifiers and deep learning algorithm for automatic grading of prostate cancer in HI on their new CAD system. Specifically, they have evaluated the performance of SVM, random forest with several number of trees, logistic regression, and linear discriminant analysis, and they also estimated the performance of a convolutional neural network (CNN) on the same training and testing subsets. They used Cohen’s kappa coefficient to evaluate the performance. The highest value attained is 0.52 by logistic regression, while 0.37 is attained by using CNN. More recently, the authors in [113] tried to compare different architectures of CNN—EfficientNet, DenseNet, and U-Net—on two datasets of prostate cancer HI. Experiments were performed on three-fold cross-validation and U-Net attained the best results.

Some researchers have studied on the use of DL techniques for automated segmentation of prostate cancer on histopathology images. In [25], the authors tried to overcome the struggles of CNN to distinguish overlapping segmentation instances. The study presented a new nuclei segmentation technique that utilized the conditional generative adversarial network (cGAN). Their proposed technique enforces a higher consistency when compared with traditional CNN architectures. In [26], the authors proposed a new nuclei boundary (NB) segmentation technique using CNN. The technique was proved to be efficient and faster than other traditional techniques, as one image of dimension 1000 × 1000 pixel can be segmented in less than five seconds. It works in the following way: firstly, the images are normalized into the same color space. Secondly, images are split into overlapping patches to tackle the extremely large image challenge. Thirdly, they proposed a new nucleus segmentation technique to identify nuclei and boundaries on each patch. Finally, the predictions of all the patches are combined to get the final prediction result of the whole image. Driven by the success of region-based CNN (RCNN) and its extensions, authors in [24] applied RCNN for detection epithelial cells employing grading network head (GNH). They applied a ResNet in their network for feature selection. Then, they employed GNH for detecting the class. They added a branch that produces an epithelial cell score using GNH. Since the proposed network was inspired by Mask RCNN, it was named Path R-CNN. The details regarding deep learning methods for prostate histopathology images have been summarized in Table 5.

## 4. Conclusions and Future Perspectives

More than 28% of cancers in men arise in the prostate gland, causing prostate cancer, and detection of this type has a high priority in cancer research. Histopathology images may enhance the early diagnosis and treatment of prostate cancer patients through providing functional and morphological data about the prostate. Histology is nothing but examining the stained sample on the slide glass under a microscope. In this survey, we presented a literature review of the use of histopathology images and its challenges. We studied different steps of histopathology image analysis methodology. This automatic process assists pathologists and clinicians in diagnosis and lowers the time spent for examining large number of tissues. The survey revealed a greater utilization of deep learning techniques and a constant use of conventional machine learning techniques. It also revealed that the histopathology image analysis is a topic of increasing interest. Our findings reveal that there is still room for improvement as CAD systems of histology images composed of complicated combination of image processing, feature selection, image segmentation, and classification stage. Moreover, the image processing techniques mentioned in this survey is not applicable for prostate histopathology image analysis only, but also applicable in many image analysis domains. This research is an attempt to summarize the most common and recent developments in prostate cancer CAD systems using histopathology images and to give an outline on the performance and efficacy of different techniques.

The domain of histopathology image processing of prostate cancer detection is very vast. According to the challenges to this type of images and disease characteristics, research in this domain is still being unlocked and many opportunities and future perspectives remain to study and analyze including: (I) the ability of enhanced interaction with images from various scanners and across pathologies, in addition to the development of new techniques that can learn from unlabeled or weakly labeled data; (II) allowing online consultations; (III) providing accessible histopathology analysis services in remote areas with limited pathology assist; (IV) developing of new data fusion techniques for integrating radiologic and histologic measurements for improved disease diagnosis with the functionality of real-time image processing and finally (V) applications and computerized software for histopathological image processing techniques may be incorporated into microscopes with small size chips. It is therefore expected from those opportunities and future perspective that we are standing at the threshold of an era that will transform the personalized diagnosis into better diagnostic systems to decrease the workload of pathologists.

## Figures and Tables

**Table 1:** A brief comparison between previous studies that proposed techniques for prostate histopathology images.

Reference	Study Aim	Year	Strength	Weakness	Number of Patients
[2]	Automated classification using AdaBoost-based Ensemble Learning	2016	They integrated various feature descriptors, different color channels, and classifiers.	The algorithm able to discover only the critical regions on the digital slides	50
[14]	A novel technique of labeling individual glands as malignant or benign was proposed.	2013	The technique can detect individual malignant gland units without relying on the neighboring histology and/or the spatial extent of the cancer.	It applied on a small number of radical prostatectomy patients	8
[15]	Methodology for automated gland and nuclei segmentation	2008	They incorporate low-, high-level knowledge, and structural constraints imposed via domain knowledge.	They focused on a smaller cohort of cancer images and the dataset is private	44
[16]	A new automated method for gland segmentation	2017	This method texture- and gland structure-based methods	The method failed in the images with the cribriform pattern. They validated data using 2-fold cross validation	10
[17]	Multistage Segmentation Using Sample Entropy Texture Analysis	2020	An added advantage of performing multistage segmentation using sample entropy values is that one could easily separate epithelial nuclei from the stroma nuclei in standard H&E stained images without using any additional immunohistochemical (IHC) markers.	It requires identifying sample entropy features	25
[18]	A new approach to identify prostate cancer areas in complex	2014	It utilizes the differential information embedded in the intensity characteristics of H&E images to quickly classify areas of the prostate tissue	Classification performance is tested using only KNN algorithm	20
[19]	Ensemble based system for feature selection and classification	2011	They addressed the possibility of missing tumor regions through the use of tile-based probabilities and heat maps.	They focused only on texture feature selection and not used a voting schema for the ensemble classifier to enhance the probability scores	14
[20]	A novel fully automated CAD system	2006	The proposed system represents the first attempt to automatically analyse histopathology across multiple scales	Their system trained using only 3 images	6
[21]	A new multiclass approach	2018	It obtained improved grading results	It was evaluated based on its impact on the performance of the ensemble framework only	213
[22]	A bag-of-words approach to classify images using SpeededUp Robust Features (SURF)	2016	The drawbacks of scale-invariant feature transform descriptor is overcome by the SURF descriptors causing an enhanced output accuracy	More features needed to be integrated with their feature extraction process to enhance accuracy of the classification	75
[23]	An automatic method for segmentation and classification (Integration of Salp Swarm Optimization Algorithm and Rider Optimization Algorithm)	2019	Less time complexity	The maximal accuracy, sensitivity, and specificity does not exceed 90%	20
[24]	A new region-based convolutional neural network framework for multi-task prediction	2018	The model achieved a detection accuracy 99.07% with an average area under the curve of 0.998	They didn’t have patient-level information with which to perform a more rigorous patient-level stratification.	40
[25]	An approach to nuclei segmentation using a conditional generative adversarial network	2019	It enforces higher-order consistency and captures better results when compared to conventional CNN models.	The model trained on small annotated patches	34
[26]	Deep neural network algorithm for segmentation of individual nuclei	2019	A simple, fast, and parameter-free postprocessing procedure is done to get the final segmented nuclei as one 1000 × 1000 image can be segmented in less than 5 s.	The model is trained on a small number of images and has been tested on the images that may have different appearances	30
[27]	Two novel approaches (combination of 4 types of feature descriptors, advanced machine-learning classifiers) to automatically identify prostate cancer	2019	They apply for the first time on prostate segmented glands, deep-learning algorithms modifying the popular VGG19 neural network.	The hand-driven learning approach employs SVM, where selecting the suitable kernel function could be tricky	35
[28]	Automated Gleason grading via deep learning	2018	The study showed promising results especially for cases with heterogeneous Gleason patterns	The model trained on small mini patches at each iteration	886
[29]	A deep learning system using the U-Net	2019	The system outperformed 10 out of 15 pathologists	The system was built upon three pretrained preprocessing modules, each of which still required pixel-wise annotations.	1243
[30]	Predicting Gleason Score Using OverFeat Trained Deep CNN as feature extractor	2016	It is quite effective, even without from-scratch training on WSI tiles. Processing time is low	Small size of patches	213
[31]	CNN to idiomatically identify the features	2016	The system is not constrained to H&E stained images and could easily be applied to immunohistochemistry	Some detection errors happen at the boundaries of the tissue	254
[32]	DL model to detect cancer based on NASNetLarge architecture and high-quality annotated training dataset	2020	The model demonstrated its strong ability in prediction as accuracy attained 98%	The availability of fully digitalized cohorts represents a bottleneck	400
[33]	A novel benchmark was designed for measuring and comparing the performances of different CNN models with the proposed PROMETEO	2021	Average processing time is less compared to other architectures	The network validated on 3-fold cross-validation method	470
[34]	Novel features that include spatial inter-nuclei statistics and intra-nuclei properties for discriminating high-grade prostate cancer patterns	2018	The system tackled the inter-observer variability in prostate grading and can lead to a consensus-based training that improves both classification	lack examples of the highest grades of disease	56

**Table 2:** Details of publicly available datasets containing prostate histopathology images.

Dataset	URL	Magnification	Year	Dataset Size	Number of Patients
Annotated dataset	[75]	40×	2017	4 images for training and 2 for validation	6
Prostate Fused-MRI-Pathology	[76]	20×	Last modified 2021	comprises a total of 28 3 Tesla T1-weighted, T2-weighted, Diffusion weighted and Dynamic Contrast Enhanced prostate MRI along with accompanying digitized histopathology images	28
TCGA-PRAD project	[77]	40×	Last modified 2020	It includes includes 368 digitized prostate pathology slides	14
Prostate cANcer graDe Assessment (PANDA) Challenge	[78]	20×	2020	It consists of 11.000 cases for training, 400 cases for public test set, and 400 cases for private test set	NA
PESO dataset	[79]	10×	2019	It consists of 62 case for the training set and 40 case for the testing set	102

**Table 3:** Summary of publications focused on feature selection of prostate histopathology images.

Features Type	Reference	Year	Accuracy Result
Texture	[56]	2011	The AUC value is 0.91 for the first database and 0.96 for the second database.
	[102]	2015	The proposed method outperforms the classic SVM-RFE in accuracy and reducing redundancy.
	[103]	2018	The proposed method attained a classification accuracy around 99%.
Topological	[13]	2011	The model attainted an average accuracy 90%.
	[50]	2011	The test classification results have an average of 96.76%
	[49]	2017	The developed way achieved 93.0% training accuracy and 97.6% testing accuracy, for the tested cases.
Morphological	[15]	2007	Average accuracy for prostate cancer classification was 92.48%
	[104]	2011	The system achieved 0.55 under the precision recall curve measure
	[58]	2019	The prediction model resulted an average accuracy of 90.2%
Color	[98]	2012	The proposed method attained an average of 86% accuracy in classifying a tissue pattern into different classes.
	[105]	2006	They achieved accuracy of 91.3%
Color & Texture	[106]	2012	The algorithm achieved an average of 86% and 93% of classification accuracy.
	[107]	2012	Classification accuracies are 97.6%, 96.6% and 87.3% when differentiating Gleason 4 versus Gleason 3, Gleason 5 versus Gleason 3, and Gleason 5 versus Gleason 4.
Topological & Morphological & Texture	[48]	2007	SVM classifier applied to test the accuracy of the extracted features and achieved about 93% when differentiating among Gleason grade 3 and stroma, 92.4% among epithelium and stroma, and 76.9% among Gleason 4 and 3.
	[27]	2019	The proposed model using hand-crafted features achieved an average accuracy of 94.6%.

**Table 4:** Summary of publications focused on Prostate histopathology image classification.

Classifier	Reference	Year	AUC	Accuracy	Specificity	Sensitivity
KNN	[66]	2003	-	0.917	-	-
	[18]	2014	-	0.76	-	-
SVM	[48]	2007	-	0.876	-	-
	[14]	2013	0.75	-	0.83	0.81
	[13]	2019	0.98 ± 0.011 for artefacts versus glands 0.92 ± 0.04 for benign versus pathological	0.95 ± 0.02 for artefacts versus glands 0.88 ± 0.07 for benign versus pathological	0.95 ± 0.03 for artefacts versus glands 0.87 ± 0.07 for benign versus pathological	0.94 ± 0.01 for artefacts versus glands 0.80 ± 0.06 for benign versus pathological
	[58]	2019	-	0.655 (one-shot classification) 0.92 (Binary classification)	-	-
Bag-of-Words	[22]	2016	-	0.901	0.905	0.79
MLA	[21]	2018	-	0.883	0.94	0.876
Boosting Cascade	[20]	2006	-	0.88	-	-
SVM and Random Forest	[19]	2011	0.95	-	0.91	0.89
Fuzzy Set Theory + Genetic Algorithm	[110]	2013	0.824	-	0.95714	0.7097
Adaboost	[2]	2016	-	0.978	-	-

**Table 5:** Summary of publications focused on applying deep learning methods for prostate histopathology images.

Method			Reference	Year	Accuracy Result	Software
CNN			[31]	2016	AUC ranges from 0.88 to 0.99.	N/A
CNN built upon VGG19			[27]	2019	Average accuracy of classifying Artefacts vs. Glands is 95.4%, average accuracy of classifying Benign vs. Pathological is 88.3%, Average accuracy of Multi-class classification is 87.6%	Matlab 2018b + Python 3.5 with Keras library and Tensorflow as backend.
Pretrained CNN			[30]	2016	The classification accuracy per image patch is 81%, while for the whole images, the classification accuracy is 89%.	N/A
Different CNN Architectures	ResNet-50		[28]	2018	They evaluated their results using test cohort and they observed that MobileNet attained the best performance on the validation set	Python 3 with Keras library and tensorflow as backend. Some analysis was done in R by the help of using survminer and survival packages.
	MobileNet					
	Inception-V3					
	DenseNet-121					
	VGG-16					
U-Net			[29]	2020	The developed model achieved accuracy of 99% for biopsies containing tumor and a specificity of 82%.	Tensorflow and Keras
SSA-RideNN			[23]	2019	The technique achieved maximal accuracy of 89.6% and sensitivity of 89.1%, and specificity of 85.9%	Matlab
SVM			[34]	2018	They used Cohen’s kappa coefficient to evaluate the performance. The highest value attained is 0.52 by logistic regression, while 0.37 is attained by using CNN.	Matlab
Random forest						
linear discriminant analysis						
logistic regression						
CNN						
Different CNN Architectures		EfficientNet	[113]	2020	UNet attained the best result of AUC about 0.98	N/A
		DenseNet				
		U-Net				
cGAN			[25]	2018	The proposed technique achieved F1-score 85.7% for prostate dataset	Pytorch 0.4
NB that utilizes CNN			[26]	2019	Their proposed model achieves 81.3% precision, 91.4% in recall, and 85.4% in F1.	Python 2.7 with Keras library and Tensorflow
Path RCNN			[24]	2019	Path RCNN attained accuracy of 99% and a mean of area under the curve of 0.99.	Python and Tensorflow backend

## Data Availability

No data available.

## References

[1] Harmon S.A., Tuncer S., Sanford T., Choyke P.L., Turkbey B. (2019). Artificial intelligence at the intersection of pathology and radiology in prostate cancer. Diagn. Interv. Radiol..

[2] Huang C.-H., Kalaw E.M. Automated classification for pathological prostate images using AdaBoost-based Ensemble Learning. Proceedings of the 2016 IEEE Symposium Series on Computational Intelligence (SSCI).

[3] Reda I., Ayinde B.O., Elmogy M., Shalaby A., El-Melegy M., El-Ghar M.A., El-Fetouh A.A., Ghazal M., El-Baz A. A new CNN-based system for early diagnosis of prostate cancer. Proceedings of the 2018 IEEE 15th International Symposium on Biomedical Imaging (ISBI 2018).

[4] Ried K., Tamanna T., Matthews S., Eng P., Sali A. (2020). New Screening Test Improves Detection of Prostate Cancer Using Circulating Tumor Cells and Prostate-Specific Markers. Front. Oncol..

[5] American Cancer Society Key Statistics for Prostate Cancer. http://www.cancer.org/cancer/prostate-cancer/about/key-statistics.html.

[6] Hoogland A.M., Kweldam C.F., Van Leenders G.J.L.H. (2014). Prognostic Histopathological and Molecular Markers on Prostate Cancer Needle-Biopsies: A Review. BioMed Res. Int..

[7] de Matos J., Britto A.D.S., Oliveira L.E., Koerich A.L. (2019). Histopathologic image processing: A review. arXiv.

[8] Komura D., Ishikawa S. (2018). Machine Learning Methods for Histopathological Image Analysis. Comput. Struct. Biotechnol. J..

[9] Aswathy M., Jagannath M. (2017). Detection of breast cancer on digital histopathology images: Present status and future possibilities. Inform. Med. Unlocked.

[10] Wang S., Burtt K., Turkbey B., Choyke P., Summers R.M. (2014). Computer Aided-Diagnosis of Prostate Cancer on Multiparametric MRI: A Technical Review of Current Research. BioMed Res. Int..

[11] Anuranjeeta, Shukla K.K., Tiwari A., Sharma S. (2017). Classification of Histopathological Images of Breast Cancerous and Non Cancerous Cells based on Morphological Features. Biomed. Pharmacol. J..

[12] Serag A., Ion-Margineanu A., Qureshi H., McMillan R., Saint Martin M.J., Diamond J., O’Reilly P., Hamilton P. (2019). Translational AI and Deep Learning in Diagnostic Pathology. Front. Med..

[13] Madabhushi A., Agner S., Basavanhally A., Doyle S., Lee G. (2011). Computer-aided prognosis: Predicting patient and disease outcome via quantitative fusion of multi-scale, multi-modal data. Comput. Med. Imaging Graph..

[14] Rashid S., Fazli L., Boag A., Siemens R., Abolmaesumi P., Salcudean S.E. (2013). Separation of Benign and Malignant Glands in Prostatic Adenocarcinoma. Proceedings of the International Conference on Medical Image Computing and Computer-Assisted Intervention.

[15] Naik S., Doyle S., Agner S., Madabhushi A., Feldman M., Tomaszewski J. Automated gland and nuclei segmentation for grading of prostate and breast cancer histopathology. Proceedings of the 2008 5th IEEE International Symposium on Biomedical Imaging: From Nano to Macro.

[16] Singh M., Kalaw E.M., Giron D.M., Chong K.-T., Tan C.L., Lee H.K. (2017). Gland segmentation in prostate histopathological images. J. Med. Imaging.

[17] Ali T., Masood K., Irfan M., Draz U., Nagra A., Asif M., Alshehri B., Glowacz A., Tadeusiewicz R., Mahnashi M. (2020). Multistage Segmentation of Prostate Cancer Tissues Using Sample Entropy Texture Analysis. Entropy.

[18] Salman S., Ma Z., Mohanty S., Bhele S., Chu Y.-T., Knudsen B., Gertych A. (2014). A Machine Learning Approach to Identify Prostate Cancer Areas in Complex Histological Images. Intelligent and Fuzzy Techniques in Big Data Analytics and Decision Making.

[19] DiFranco M.D., O’Hurley G., Kay E.W., Watson R.W.G., Cunningham P. (2011). Ensemble based system for whole-slide prostate cancer probability mapping using color texture features. Comput. Med. Imaging Graph..

[20] Doyle S., Madabhushi A., Feldman M., Tomaszeweski J. (2006). A Boosting Cascade for Automated Detection of Prostate Cancer from Digitized Histology. Proceedings of the International Conference on Medical Image Computing and Computer-Assisted Intervention.

[21] Albashish D., Sahran S., Abdullah A., Adam A., Alweshah M. (2018). A hierarchical classifier for multiclass prostate histopathology image gleason grading. J. Inf. Commun. Technol..

[22] Sanghavi F.M. (2016). Automated classification of histopathology images of prostate cancer using a Bag-of-Words approach. Mobile Multimedia/Image Processing, Security, and Applications 2016.

[23] Gurav S.B., Kulhalli K.V., Desai V.V. (2019). Prostate cancer detection using histopathology images and classification using improved RideNN. Biomed. Eng. Appl. Basis Commun..

[24] Li W., Li J., Sarma K.V., Ho K.C., Shen S., Knudsen B.S., Gertych A., Arnold C.W. (2019). Path R-CNN for Prostate Cancer Diagnosis and Gleason Grading of Histological Images. IEEE Trans. Med. Imaging.

[25] Mahmood F., Borders D., Chen R.J., McKay G.N., Salimian K.J., Baras A., Durr N.J. (2020). Deep Adversarial Training for Multi-Organ Nuclei Segmentation in Histopathology Images. IEEE Trans. Med. Imaging.

[26] Cui Y., Zhang G., Liu Z., Xiong Z., Hu J. (2019). A deep learning algorithm for one-step contour aware nuclei segmentation of histopathology images. Med. Biol. Eng. Comput..

[27] García G., Colomer A., Naranjo V. (2019). First-Stage Prostate Cancer Identification on Histopathological Images: Hand-Driven versus Automatic Learning. Entropy.

[28] Arvaniti E., Fricker K.S., Moret M., Rupp N., Hermanns T., Fankhauser C., Wey N., Wild P.J., Rüschoff J.H., Claassen M. (2018). Automated Gleason grading of prostate cancer tissue microarrays via deep learning. Sci. Rep..

[29] Bulten W., Pinckaers H., van Boven H., Vink R., de Bel T., van Ginneken B., van der Laak J., de Kaa C.H., Litjens G. (2019). Automated gleason grading of prostate biopsies using deep learning. arXiv.

[30] Kallen H., Molin J., Heyden A., Lundstrom C., Astrom K. Towards grading gleason score using generically trained deep convolutional neural networks. Proceedings of the 2016 IEEE 13th International Symposium on Biomedical Imaging (ISBI).

[31] Litjens G., Sánchez C.I., Timofeeva N., Hermsen M., Nagtegaal I., Kovacs I., Van De Kaa C.H., Bult P., Van Ginneken B., Van Der Laak J. (2016). Deep learning as a tool for increased accuracy and efficiency of histopathological diagnosis. Sci. Rep..

[32] Tolkach Y., Dohmgörgen T., Toma M., Kristiansen G. (2020). High-accuracy prostate cancer pathology using deep learning. Nat. Mach. Intell..

[33] Duran-Lopez L., Dominguez-Morales J., Rios-Navarro A., Gutierrez-Galan D., Jimenez-Fernandez A., Vicente-Diaz S., Linares-Barranco A. (2021). Performance Evaluation of Deep Learning-Based Prostate Cancer Screening Methods in Histopathological Images: Measuring the Impact of the Model’s Complexity on Its Processing Speed. Sensors.

[34] Nir G., Hor S., Karimi D., Fazli L., Skinnider B.F., Tavassoli P., Turbin D., Villamil C.F., Wang G., Wilson R.S. (2018). Automatic grading of prostate cancer in digitized histopathology images: Learning from multiple experts. Med. Image Anal..

[35] Tariq M., Iqbal S., Ayesha H., Abbas I., Ahmad K.T., Niazi M.F.K. (2021). Medical image based breast cancer diagnosis: State of the art and future directions. Expert Syst. Appl..

[36] Jimenez-del-Toro O., Otálora S., Andersson M., Eurén K., Hedlund M., Rousson M., Atzori M. (2017). Analysis of histopathology images: From traditional machine learning to deep learning. Biomedical Texture Analysis.

[37] Madabhushi A., Lee G. (2016). Image analysis and machine learning in digital pathology: Challenges and opportunities. Med. Image Anal..

[38] Belsare A. (2012). Histopathological Image Analysis Using Image Processing Techniques: An Overview. Signal Image Process. Int. J..

[39] Arevalo J., Cruz-Roa A., González F.A. (2014). Histopathology image representation for automatic analysis: A state-of-the-art review. Rev. Med..

[40] Jothi J.A.A., Rajam V.M.A. (2017). A survey on automated cancer diagnosis from histopathology images. Artif. Intell. Rev..

[41] Das A., Nair M.S., Peter S.D. (2020). Computer-Aided Histopathological Image Analysis Techniques for Automated Nuclear Atypia Scoring of Breast Cancer: A Review. J. Digit. Imaging.

[42] Madabhushi A. (2009). Digital pathology image analysis: Opportunities and challenges. Imaging Med..

[43] Humphrey P.A. (2017). Histopathology of Prostate Cancer. Cold Spring Harb. Perspect. Med..

[44] Mosquera-Lopez C., Agaian S., Velez-Hoyos A., Thompson I. (2015). Computer-Aided Prostate Cancer Diagnosis from Digitized Histopathology: A Review on Texture-Based Systems. IEEE Rev. Biomed. Eng..

[45] Li C., Chen H., Li X., Xu N., Hu Z., Xue D., Qi S., Ma H., Zhang L., Sun H. (2020). A review for cervical histopathology image analysis using machine vision approaches. Artif. Intell. Rev..

[46] Krithiga R., Geetha P. (2020). Breast Cancer Detection, Segmentation and Classification on Histopathology Images Analysis: A Systematic Review. Arch. Comput. Methods Eng..

[47] Van Booven D.J., Kuchakulla M., Pai R., Frech F.S., Ramasahayam R., Reddy P., Parmar M., Ramasamy R., Arora H. (2021). A Systematic Review of Artificial Intelligence in Prostate Cancer. Res. Rep. Urol..

[48] Doyle S., Hwang M., Shah K., Madabhushi A., Feldman M., Tomaszeweski J. Automated grading of prostate cancer using architectural and textural image features. Proceedings of the 2007 4th IEEE International Symposium on Biomedical Imaging: From Nano to Macro.

[49] Niazi M.K.K., Yao K., Zynger D.L., Clinton S.K., Chen J., Koyuturk M., LaFramboise T., Gurcan M. (2017). Visually Meaningful Histopathological Features for Automatic Grading of Prostate Cancer. IEEE J. Biomed. Health Inform..

[50] Khurd P., Bahlmann C., Maday P., Kamen A., Gibbs-Strauss S., Genega E.M., Frangioni J.V. Computer-aided Gleason grading of prostate cancer histopathological images using texton forests. Proceedings of the 2010 IEEE International Symposium on Biomedical Imaging: From Nano to Macro.

[51] Slaoui M., Fiette L. (2010). Histopathology Procedures: From Tissue Sampling to Histopathological Evaluation. Methods in Molecular Biology.

[52] Cahill L.C., Fujimoto J.G., Giacomelli M.G., Yoshitake T., Wu Y., Lin D.I., Ye H., Carrasco-Zevallos O.M., Wagner A.A., Rosen S. (2019). Comparing histologic evaluation of prostate tissue using nonlinear microscopy and paraffin H&E: A pilot study. Mod. Pathol..

[53] Xu Y., Jia Z., Wang L.-B., Ai Y., Zhang F., Lai M., Chang E.I.-C. (2017). Large scale tissue histopathology image classification, segmentation, and visualization via deep convolutional activation features. BMC Bioinform..

[54] Zangeneh E., Rahmati M., Mohsenzadeh Y. (2020). Low resolution face recognition using a two-branch deep convolutional neural network architecture. Expert Syst. Appl..

[55] Kramberger T., Potočnik B. (2020). LSUN-Stanford Car Dataset: Enhancing Large-Scale Car Image Datasets Using Deep Learning for Usage in GAN Training. Appl. Sci..

[56] Peng Y., Jiang Y., Eisengart L., Healy M.A., Straus F.H., Yang X.J. (2011). Computer-aided identification of prostatic adenocarcinoma: Segmentation of glandular structures. J. Pathol. Inform..

[57] Zhu C., Song F., Wang Y., Dong H., Guo Y., Liu J. (2019). Breast cancer histopathology image classification through assembling multiple compact CNNs. BMC Med. Inform. Decis. Mak..

[58] Bhattacharjee S., Park H.-G., Kim C.-H., Prakash D., Madusanka N., So J.-H., Cho N.-H., Choi H.-K. (2019). Quantitative Analysis of Benign and Malignant Tumors in Histopathology: Predicting Prostate Cancer Grading Using SVM. Appl. Sci..

[59] Dimitriou N., Arandjelović O., Caie P.D. (2019). Deep Learning for Whole Slide Image Analysis: An Overview. Front. Med..

[60] Veta M.M., Pluim J.P.W., Van Diest P.J., Viergever M.A. (2014). Breast Cancer Histopathology Image Analysis: A Review. IEEE Trans. Biomed. Eng..

[61] Şerbănescu M.-S., Manea N.C., Streba L., Belciug S., Pleşea I.E., Pirici I., Bungărdean R.M., Pleşea R.M. (2020). Automated Gleason grading of prostate cancer using transfer learning from general-purpose deep-learning networks. Rom. J. Morphol. Embryol. Rev. Roum. Morphol. Embryol..

[62] Arvaniti E., Claassen M. (2018). Coupling weak and strong supervision for classification of prostate cancer histopathology images. arXiv.

[63] A Sharif S.M., Naqvi R.A., Biswas M. (2020). Learning Medical Image Denoising with Deep Dynamic Residual Attention Network. Mathematics.

[64] Çelik G., Talu M.F. (2020). Resizing and cleaning of histopathological images using generative adversarial networks. Phys. A Stat. Mech. Its Appl..

[65] Arif M., Rajpoot N. Classification of potential nuclei in prostate histology images using shape manifold learning. Proceedings of the 2007 International Conference on Machine Vision.

[66] Jafari-Khouzani K., Soltanian-Zadeh H. (2003). Multiwavelet grading of pathological images of prostate. IEEE Trans. Biomed. Eng..

[67] Li X., Plataniotis K.N. (2015). A Complete Color Normalization Approach to Histopathology Images Using Color Cues Computed From Saturation-Weighted Statistics. IEEE Trans. Biomed. Eng..

[68] Piórkowski A. Color Normalization-Based Nuclei Detection in Images of Hematoxylin and Eosin-Stained Multi Organ Tissues. Proceedings of the International Conference on Image Processing and Communications.

[69] Xiao Y., Decenciere E., Velasco-Forero S., Burdin H., Bornschlogl T., Bernerd F., Warrick E., Baldeweck T. A New Color Augmentation Method for Deep Learning Segmentation of Histological Images. Proceedings of the 2019 IEEE 16th International Symposium on Biomedical Imaging (ISBI 2019).

[70] Vicory J., Couture H.D., Thomas N.E., Borland D., Marron J., Woosley J., Niethammer M. (2015). Appearance normalization of histology slides. Comput. Med. Imaging Graph..

[71] Gu Y., Yang J. (2019). Multi-level magnification correlation hashing for scalable histopathological image retrieval. Neurocomputing.

[72] Campanella G., Hanna M.G., Geneslaw L., Miraflor A., Silva V.W.K., Busam K.J., Brogi E., Reuter V.E., Klimstra D.S., Fuchs T.J. (2019). Clinical-grade computational pathology using weakly supervised deep learning on whole slide images. Nat. Med..

[73] McClure P., Elnakib A., El-Ghar M.A., Khalifa F., Soliman A., El-Diasty T., Suri J.S., Elmaghraby A., El-Baz A. (2014). In-Vitro and In-Vivo Diagnostic Techniques for Prostate Cancer: A Review. J. Biomed. Nanotechnol..

[74] Reda I., Khalil A., Elmogy M., El-Fetouh A.A., Shalaby A., El-Ghar M.A., Elmaghraby A., Ghazal M., El-Baz A. (2018). Deep Learning Role in Early Diagnosis of Prostate Cancer. Technol. Cancer Res. Treat..

[75] Kumar N., Verma R., Sharma S., Bhargava S., Vahadane A., Sethi A. (2017). A Dataset and a Technique for Generalized Nuclear Segmentation for Computational Pathology. IEEE Trans. Med. Imaging.

[76] Prostate Fused-MRI-Pathology. https://wiki.cancerimagingarchive.net/display/Public/Prostate+Fused-MRI-Pathology.

[77] TCGA-PRAD. https://wiki.cancerimagingarchive.net/display/Public/TCGA-PRAD.

[78] Prostate cANcer graDe Assessment (PANDA) Challenge. https://www.kaggle.com/c/prostate-cancer-grade-assessment/data.

[79] PESO: Prostate Epithelium Segmentation on H&E-Stained Prostatectomy Whole Slide Images. https://zenodo.org/record/1485967#.YF945q8zbIU.

[80] Jain R., Kasturi R., Schunck B.G. (1995). Machine Vision.

[81] Hoshyar A.N., Al-Jumaily A., Hoshyar A.N. (2014). The Beneficial Techniques in Preprocessing Step of Skin Cancer Detection System Comparing. Procedia Comput. Sci..

[82] Patidar P., Gupta M., Srivastava S., Nagawat A.K. (2010). Image De-noising by Various Filters for Different Noise. Int. J. Comput. Appl..

[83] Lee G., Singanamalli A., Wang H., Feldman M.D., Master S.R., Shih N.N.C., Spangler E., Rebbeck T., Tomaszewski J.E., Madabhushi A. (2014). Supervised multi-view canonical correlation analysis (sMVCCA): Integrating histologic and proteomic features for predicting recurrent prostate cancer. IEEE Trans. Med Imaging.

[84] Gurcan M.N., Boucheron L.E., Can A., Madabhushi A., Rajpoot N.M., Yener B. (2009). Histopathological Image Analysis: A Review. IEEE Rev. Biomed. Eng..

[85] Yang L., Meer P., Foran D.J. (2005). Unsupervised segmentation based on robust estimation and color active contour models. IEEE Trans. Inf. Technol. Biomed..

[86] Bautista P.A., Hashimoto N., Yagi Y. (2014). Color standardization in whole slide imaging using a color calibration slide. J. Pathol. Inform..

[87] Zuo C., Chen Q., Sui X. (2013). Range Limited Bi-Histogram Equalization for image contrast enhancement. Optik.

[88] Tam A., Barker J., Rubin D.L. (2016). A method for normalizing pathology images to improve feature extraction for quantitative pathology. Med. Phys..

[89] Shanmugavadivu P., Balasubramanian K. (2014). Particle swarm optimized multi-objective histogram equalization for image enhancement. Opt. Laser Technol..

[90] Nanni L., Brahnam S., Ghidoni S., Maguolo G. (2019). General purpose (GenP) bioimage ensemble of handcrafted and learned features with data augmentation. arXiv.

[91] Shin H.-C., Tenenholtz N.A., Rogers J.K., Schwarz C.G., Senjem M.L., Gunter J.L., Andriole K.P., Michalski M. (2018). Medical Image Synthesis for Data Augmentation and Anonymization Using Generative Adversarial Networks. Tools and Algorithms for the Construction and Analysis of Systems.

[92] Sandfort V., Yan K., Pickhardt P.J., Summers R.M. (2019). Data augmentation using generative adversarial networks (CycleGAN) to improve generalizability in CT segmentation tasks. Sci. Rep..

[93] Liu S., Shah Z., Sav A., Russo C., Berkovsky S., Qian Y., Coiera E., Di Ieva A. (2020). Isocitrate dehydrogenase (IDH) status prediction in histopathology images of gliomas using deep learning. Sci. Rep..

[94] Ataky S.T.M., De Matos J., Britto A.D.S., Oliveira L.E.S., Koerich A.L. Data Augmentation for Histopathological Images Based on Gaussian-Laplacian Pyramid Blending. Proceedings of the 2020 International Joint Conference on Neural Networks (IJCNN).

[95] Chauhan N.K., Singh K. A Review on Conventional Machine Learning vs Deep Learning. Proceedings of the 2018 International Conference on Computing, Power and Communication Technologies (GUCON).

[96] Nielsen B., Albregtsen F., Danielsen H.E. (2012). Automatic segmentation of cell nuclei in Feulgen-stained histological sections of prostate cancer and quantitative evaluation of segmentation results. Cytom. Part A.

[97] Simon I., Pound C.R., Partin A.W., Clemens J.Q., Christens-Barry W.A. (1998). Automated image analysis system for detecting boundaries of live prostate cancer cells. Cytometry.

[98] Nguyen K., Sabata B., Jain A.K. (2012). Prostate cancer grading: Gland segmentation and structural features. Pattern Recognit. Lett..

[99] Nguyen K., Jain A.K., Allen R.L. Automated Gland Segmentation and Classification for Gleason Grading of Prostate Tissue Images. Proceedings of the 2010 20th International Conference on Pattern Recognition.

[100] Ayyad S.M., Saleh A.I., Labib L.M., Aiyad S.M. (2019). A new distributed feature selection technique for classifying gene expression data. Int. J. Biomath..

[101] Ayyad S.M., Saleh A.I., Labib L.M. (2019). Gene expression cancer classification using modified K-Nearest Neighbors technique. Biosystems.

[102] Albashish D., Sahran S., Abdullah A., Adam A., Shukor N.A., Pauzi S.H.M. Multi-scoring feature selection method based on SVM-RFE for prostate cancer diagnosis. Proceedings of the 2015 International Conference on Electrical Engineering and Informatics (ICEEI).

[103] Peyret R., Bouridane A., Khelifi F., Tahir M.A., Al-Maadeed S. (2018). Automatic classification of colorectal and prostatic histologic tumor images using multiscale multispectral local binary pattern texture features and stacked generalization. Neurocomputing.

[104] Sparks R., Madabhushi A. (2011). Content-based image retrieval utilizing explicit shape descriptors: Applications to breast MRI and prostate histopathology. SPIE Med. Imaging.

[105] Tabesh A., Teverovskiy M. Tumor Classification in Histological Images of Prostate Using Color Texture. Proceedings of the 2006 Fortieth Asilomar Conference on Signals, Systems and Computers.

[106] Akakin H.C., Gurcan M.N. (2012). Content-Based Microscopic Image Retrieval System for Multi-Image Queries. IEEE Trans. Inf. Technol. Biomed..

[107] Lopez C.M., Agaian S., Sanchez I., Almuntashri A., Zinalabdin O., Al Rikabi A., Thompson I. Exploration of efficacy of gland morphology and architectural features in prostate cancer gleason grading. Proceedings of the 2012 IEEE International Conference on Systems, Man, and Cybernetics (SMC).

[108] Shaban W.M., Rabie A.H., Saleh A.I., Abo-Elsoud M. (2020). A new COVID-19 Patients Detection Strategy (CPDS) based on hybrid feature selection and enhanced KNN classifier. Knowl. -Based Syst..

[109] Ayyad S.M., Saleh A.I., Labib L.M. (2018). Classification techniques in gene expression microarray data. Int. J. Comput. Sci. Mob. Comput..

[110] Castanho M., Hernandes F., De Ré A., Rautenberg S., Billis A. (2013). Fuzzy expert system for predicting pathological stage of prostate cancer. Expert Syst. Appl..

[111] Shaban W.M., Rabie A.H., Saleh A.I., Abo-Elsoud M. (2020). Detecting COVID-19 patients based on fuzzy inference engine and Deep Neural Network. Appl. Soft Comput..

[112] Khan S., Yong S.-P. A comparison of deep learning and hand crafted features in medical image modality classification. Proceedings of the 2016 3rd International Conference on Computer and Information Sciences (ICCOINS).

[113] Swiderska-Chadaj Z., De Bel T., Blanchet L., Baidoshvili A., Vossen D., Van Der Laak J., Litjens G. (2020). Impact of rescanning and normalization on convolutional neural network performance in multi-center, whole-slide classification of prostate cancer. Sci. Rep..

